# Treatment intensity and outcome of nonagenarians selected for admission in ICUs: a multicenter study of the Outcomerea Research Group

**DOI:** 10.1186/s13613-016-0133-9

**Published:** 2016-04-14

**Authors:** Maité Garrouste-Orgeas, Stéphane Ruckly, Charles Grégoire, Anne-Sylvie Dumesnil, Cécile Pommier, Samir Jamali, Dany Golgran-Toledano, Carole Schwebel, Christophe Clec’h, Lilia Soufir, Muriel Fartoukh, Guillaume Marcotte, Laurent Argaud, Bruno Verdière, Michael Darmon, Elie Azoulay, Jean-François Timsit

**Affiliations:** Service de Réanimation et de médecine intensive, Medical-Surgical ICU, Saint Joseph Hospital Network, 185 rue Raymond Losserand, 75014 Paris, France; Infection, Antimicrobials, Modelling, Evolution (IAME), UMR 1137, INSERM and Paris Diderot University, Department of Biostatistics - HUPNVS. - AP-HP, UFR de Médecine, Bichat University Hospital, Paris, France; Department of Biostatistics, Outcomerea, Paris, France; Medical-Surgical ICU, AP-HP, Antoine Béclère University Hospital, Clamart, France; Medical-Surgical, General Hospital, Dourdan, France; Medical-Surgical ICU, General Hospital, Montfermeil, France; Medical ICU, Albert Michallon University Hospital, Grenoble, France; Medical-Surgical ICU, AP-HP, Avicennes University Hospital, Bobigny, France; Medical ICU, AP-HP, Tenon University Hospital, Paris, France; Medical-Surgical ICU, Hospices civils de Lyon, Hôpital Edouard Herriot, Lyon, France; Medical ICU, Hospices civils de Lyon, Hôpital Edouard Herriot, Lyon, France; Medical-Surgical ICU, Delafontaine University Hospital, Saint Denis, France; Medical-Surgical ICU, Saint Etienne University Hospital, Saint Priest en Jarez, France; Medical ICU, AP-HP, Saint Louis University Hospital, Paris, France; Medical ICU, AP-HP, Bichat University Hospital, Paris, France

**Keywords:** Elderly, 80 and over, Intensive care unit, End of life, Triage

## Abstract

**Background:**

Outcome of very elderly patients admitted in intensive care unit (ICU) was most often reported for octogenarians. ICU admission demands for nonagenarians are increasing. The primary objective was to compare outcome and intensity of treatment of octogenarians and nonagenarians.

**Methods:**

We performed an observational study in 12 ICUs of the Outcomerea™ network which prospectively upload data into the Outcomerea™ database. Patients >90 years old (case patients) were matched with patients 80–90 years old (control patients). Matching criteria were severity of illness at admission, center, and year of admission.

**Results:**

A total of 2419 patients aged 80 or older and admitted from September 1997 to September 2013 were included. Among them, 179 (7.9 %) were >90 years old. Matching was performed for 176 nonagenarian patients. Compared with control patients, case patients were more often hospitalized for unscheduled surgery [54 (30.7 %) vs. 42 (23.9 %), *p* < 0.01] and had less often arterial monitoring for blood pressure [37 (21 %) vs. 53 (30.1 %), *p* = 0.04] and renal replacement therapy [5 (2.8 %) vs. 14 (8 %), *p* = 0.05] than control patients. ICU [44 (25 %) vs. 36 (20.5 %), *p* = 0.28] or hospital mortality [70 (39.8 %) vs. 64 (36.4 %), *p* = 0.46] and limitation of life-sustaining therapies were not significantly different in case versus control patients, respectively. Only 16/176 (14 %) of case patients were transferred to a geriatric unit.

**Conclusion:**

This multicenter study reported that nonagenarians represented a small fraction of ICU patients. When admitted, these highly selected patients received similar life-sustaining treatments, except RRT, than octogenarians. ICU and hospital mortality were similar between the two groups.

**Electronic supplementary material:**

The online version of this article (doi:10.1186/s13613-016-0133-9) contains supplementary material, which is available to authorized users.

## Background

With population aging and medical management progress, the demands for intensive care unit admission for very elderly patients are increasing throughout the world [1]. Against this challenge, the resources for intensive care are not adapted. The intensive care unit physician has to make the complex decision which is to admit elderly patients with benefit to admission [2]. This decision may vary broadly according to countries and to physicians characteristics [3]. This decision is even more difficult with very elderly for whom advance directives are often not available [3].

Outcome of very elderly patients was most often reported for octogenarians [4]. The mean age in studies was roughly 84 years old [5–8]. Outcome was very different for patients admitted after planned surgery and patients admitted for medical reasons or unscheduled surgery. After scheduled surgery, the ICU and hospital mortality rate were 12 and 25 %, respectively [9]. For the same endpoints, rates were 38 and 64 % for medical patients and 45 and 55 % for unscheduled surgery patients [5, 6]. This known high mortality rate for medical very elderly patients is probably responsible for the identification of age >85 years as an independent factor for ICU refusal for some physicians [10, 11]. Decision of ICU admission of very elderly patients should be a shared decision taking into account the point of view of the physician and the patients. Patients are less favorable than intensivist to ICU admission [11, 12].

There are no valuable reasons to limit ICU admission according to age. ICU admission requests for nonagenarians are increasing. However, only few studies evaluated their outcome. In a Brazilian study of nonagenarians admitted in a single center, the ICU and 6-month mortality for medical patients were 38 and 47 %, respectively. Of note, 25 % of patients were mechanically ventilated [13]. In a Greek single study, nonagenarians represented 1.1 % of the admitted population. Their ICU and hospital mortality were 20 and 40 %, respectively [14]. A case–control study from France reported no significant differences in outcome in patients aged 90 and more compared with younger after adjustment on severity of illness and support of life threatening therapies [15]. These scarce studies revealed that outcomes of nonagenarians were not worse than those of octogenarians. To precise outcome of the very elderly ICU population, we conducted an observational study on very elderly patients within the French multicenter Outcomerea database, in order to compare the outcome and intensity of treatment of youngest patients (80–89 years old) versus oldest (>90 years old).

## Methods

### Study design and data collection

Data of a convenience sample of patients more than 80 years old were obtained from September 1997 to September 2013 in 12 ICUs on the multicenter database Outcomerea™.

Briefly, every year, each participating ICU enters prospective data on daily disease severity and intensity of care for at least 50 consecutive patients admitted over a 1-month period or admitted to a predefined number of beds throughout the year, as described elsewhere [16]. None of the 12 ICUs had written guidelines for ICU admission. ICU admission was guided by both intensivist’s evaluations of the appropriateness of ICU admission, patients’ preferences if available, and the availability of beds [3].

The following information was collected for each patient: age, sex, body mass index, admission category (medical, scheduled surgery, or unscheduled surgery), pre-defined admission diagnosis (multiorgan failure and shock, acute renal failure, acute respiratory failure, chronic obstructive pulmonary disease, monitoring, trauma), invasive procedures (arterial and/or venous central lines, endotracheal and noninvasive ventilation, tracheotomy, and dialysis), and treatment of organ failures [inotropic support, mechanical and noninvasive ventilation, and renal replacement therapy (RRT)]. Severity of chronic illness was measured with the Knaus classification [17]. Severity of illness was measured daily using the Simplified Acute Physiology Score II [18] and SOFA score [19, 20]. We recorded treatment limitations at ICU admission in three categories: withdrawing (decision to stop life-sustaining intervention), withholding (decision not to start or increase life-sustaining intervention when there is a need), or advance withholding (no escalation of treatment before this need arises) [21]. Vital status at ICU and hospital discharge was recorded. Transfer of the nonagenarians to an acute geriatric unit was collected after ICU discharge.

### Quality of the database

For most of the study variables, the data-capture software immediately ran an automatic check for internal consistency, generating queries that were sent to the ICUs for resolution before incorporation of the new data into the database. In each participating ICU, data quality was checked by having a senior physician from another participating ICU review a 2 % random sample of the study data every other year. A 1-day data-capture training course held once a year was open to all Outcomerea™ investigators and study monitors.

### Ethics committee approval and informed consent

According to French law on non-interventional studies, the study was approved by the Clermont-Ferrand Hospital institutional review board (CECIC, IRB #5891, ref #2007-16) and by the Pitié-Salpêtrière Hospital Ethics Committee, which waived the need for informed consent for patients included in the database. The database was disclosed to the French Data Protection Authority (CNIL 1675804 v 0).

### Statistical analysis

Data are expressed as median [interquartile range] for continuous variables and number (%) for categorical variables. Matching criteria were severity of illness, center, and year. Severity of illness was rated using corrected SAPS II age. Comparisons of matched pairs were made using McNemar’s paired test, Friedman’s test, and Wilcoxon’s paired test, as appropriate. Comparisons between 80–90 and >90 years old on patients who died in the ICU were made using Chi-square test for categorical variables and Mann–Whitney for continuous variables. All statistical analyses were carried out with SAS version 9.4 (SAS Institute Inc., Cary, NC, USA). *p* values <0.05 were considered significant.

## Results

From September 1997 to September 2013, 2,419 patients aged 80 years and over and hospitalized in 12 ICUs were included in the database. Among them, 179 (7.9 %) were >90 years old and represented the case patients. No significant increase in the number of nonagenarians ICU admissions was reported in our database during the study period (Additional file 1: Figure S1). We matched 176 case patients with 176 control patients aged between 80 and 90. Table 1 reports the demographics and clinical characteristics of the case and controls patients. Compared with control patients, with the same severity of illness at ICU admission, case patients were more often admitted for unscheduled surgery, had similar intensity of treatment (invasive and noninvasive ventilation, and use of vasopressive therapy), but had a lower use of arterial catheter monitoring (37/176, 21 % vs. 53/176, 30 %, *p* = 0.04) or RRT (5/176, 2.8 % vs. 14/176, 8 %, *p* = 0.05). Overuse of urinary catheter was seen in case patients (Table 2). The percentage of life-sustaining therapies within 48 h of ICU admission was similar in both groups. ICU and hospital length of stay were significantly shorter in case patients, while no differences in ICU or hospital mortality were reported (Table 2). Beside age, the non-survivors of both groups were not significantly different, except that case patients had shorter pre-admission length of stay and lower use of central venous and arterial catheters (Table 3).

**Table 1:** Comparison between characteristics of octogenarian patients (80–90) and matched nonagenarian patients (>90)

Variables	80–90 years old *N* = 176	>90 years old *N* = 176	*p* value^a^
Age in years, median (IQR)	83.8 (81.4–85.7)	92 (90.8–93.7)	<0.01
Males, *n* (%)	84 (47.7)	66 (37.5)	0.05
Body mass index, kg/m^2^, median (IQR)^b^	25 (22.6–28.9)	23.1 (20.4–26.1)	<0.01
Transfer from ward, *n* (%)	103 (58.5)	86 (48.9)	0.07
At least one underlying disease^c^, *n* (%)	74 (42.0)	76 (43.2)	0.83
Hepatic	3 (1.7)	3 (1.7)	1.00
Cardiovascular	43 (24.4)	55 (31.3)	0.15
Pulmonary	30 (17)	19 (10.8)	0.09
Renal	8 (4.5)	6 (3.4)	0.59
Immunosuppression	5 (2.8)	8 (4.5)	0.40
Admission category, *n* (%)			0.35
Medicine	122 (69.3)	112 (63.6)	
Unscheduled surgery	42 (23.9)	54 (30.7)	
Scheduled surgery	12 (6.8)	10 (5.7)	
Main symptom at admission, *n* (%)			
Shock and multiorgan failure	52 (29.5)	51 (29)	0.91
Acute respiratory failure	63 (35.8)	54 (30.7)	0.31
Acute renal failure	8 (4.5)	7 (4)	0.79
Coma	14 (8)	10 (5.7)	0.40
Monitoring	24 (13.6)	37 (21)	0.07
Trauma	2 (1.1)	2 (1.1)	1.00
Severity of illness at admission, median (IQR)			
SAPS II at admission	45 (37–57)	46 (37–59)	0.77
SAPS II leaving out age points	27 (19–39)	28 (19–41)	0.77
SOFA within 48 h of admission	5 (3–8)	5 (3–8)	0.96

*IQR* inter quartile range, *SAPS II* Simplified Acute Physiologic Score II, *SOFA* Sepsis Organ Failure Assessment, *ICU* intensive care unit

^a^Chi-square or Mann–Whitney test as appropriate

^b^109 missing values

^c^Defined by Knaus definition [17]

**Table 2:** Intensity of care and outcome of octogenarian patients (80–90) and matched nonagenarian patients (>90)

Variables	80–90 years old *N* = 176	>90 years old *N* = 176	*p* value
Intensity of care within 48 h of admission, *n* (%)			
Endotracheal mechanical ventilation	87 (49.4)	76 (43.2)	0.18
Noninvasive mechanical ventilation	37 (21)	24 (13.6)	0.07
Epinephrine/norepinephrine	56 (31.8)	49 (27.8)	0.41
Dobutamine	33(18.8)	33 (18.8)	1.00
Central venous catheter	80 (45.5)	64 (36.4)	0.09
Arterial catheter	53 (30.1)	37 (21)	0.04
Renal replacement therapy	14 (8)	5 (2.8)	0.05
Urinary catheter	137 (77.8)	151 (85.8)	0.05
Pre-ICU hospital stay, days, median (IQR)	1 (0–6)	0 (0–3)	0.02
Length of ICU stay, days, median (IQR)	6 (3–12)	4 (2–7.5)	<0.01
Length of hospital stay, days, median (IQR)^a^	21 (13–37.5)	16 (8–26)	<0.01
Decision to forgo life-sustaining treatments within 48 h of admission, *n* (%)			
Advance withholding^b^	18 (10.2)	16 (9.1)	0.86
Withholding	3 (1.7)	6 (3.4)	0.51
Withdrawing	4 (2.3)	3 (1.7)	1.00
ICU mortality, *n* (%)	36 (20.5)	44 (25)	0.34
Hospital mortality, *n* (%)	64 (36.4)	70 (39.8)	0.54

*IQR* inter quartile range

^a^21 missing values

^b^Anticipated decision of no escalation of life-sustaining intervention before this need arises

**Table 3:** Comparison of characteristics of non-survivors between octogenarians (80–90) and nonagenarians (>90)

Variables	80–90 years old *N* = 36	>90 years old *N* = 44	*p* value
Age in years, median (IQR)	82.9 (81.1–85.2)	92 (91–93.5)	<0.01
Males, *n* (%)	18 (50)	18 (40.9)	0.42
Body mass index, kg/m^2^, median (IQR)^a^	24.2 (22.2–26)	22 (20–27.3)	0.24
Transfer from ward, *n* (%)	21 (58.3)	17 (38.6)	0.08
At least one underlying disease^b^, *n* (%)	12 (33.3)	20 (45.5)	0.27
Hepatic	0	1 (2.3)	0.36
Cardiovascular	7 (19.4)	15 (34.1)	0.14
Pulmonary	4 (11.1)	7 (15.9)	0.54
Renal	2 (5.6)	2 (4.5)	0.84
Immunosuppression	1 (2.8)	2 (4.5)	0.68
Admission category, *n* (%)			1.00
Medicine	27 (75)	33 (75)	
Unscheduled surgery	9 (25)	11 (25)	
Scheduled surgery	0	0	
Main symptom at admission, *n* (%)			
Shock and multiorgan failure	16 (44.4)	16 (36.4)	0.46
Acute respiratory failure	13 (36.1)	16 (36.4)	0.98
Acute renal failure	0	1 (2.3)	0.36
Coma	4 (11.1)	3 (6.8)	0.50
Monitoring	3 (8.3)	2 (4.5)	0.68
Trauma	0	1 (2.3)	0.36
Severity of illness at admission, median (IQR)			
SAPS II at admission	55.5 (45.5–76)	62 (49.5–81.5)	0.20
SAPS II leaving out age points	37.5 (27.5–58)	44 (31.5–63.5)	0.20
SOFA within 48 h of admission	7 (5–10.5)	8 (6–10)	0.41
Intensity of care within 48 h of admission, *n* (%)			
Endotracheal mechanical ventilation	27 (75)	29 (65.9)	0.38
Noninvasive mechanical ventilation	9 (25)	6 (13.6)	0.20
Epinephrine/norepinephrine	23 (63.9)	23 (52.3)	0.30
Dobutamine	11 (30.6)	14 (31.8)	0.90
Central venous catheter	23 (63.9)	18 (40.9)	0.04
Arterial catheter	18 (50)	12 (27.3)	0.04
Renal replacement therapy	1 (2.8)	2 (4.5)	0.68
Urinary catheter	30 (83.3)	36 (81.8)	0.86
Pre-ICU hospital stay, days, median (IQR)	1.5 (0–6.5)	0 (0–1)	0.03
Length of ICU stay, days, median (IQR)	8.5 (2.5–19.5)	6 (2–16)	0.17
Length of hospital stay, median (IQR)	13 (5–24)	8 (3–18.5)	0.09
Decision to forgo life-sustaining treatments within 48 h of admission, *n* (%)			
Advance withholding^c^	11 (30.6)	8 (18.2)	0.20
Withholding	2 (5.6)	5 (11.4)	0.36
Withdrawing	2 (5.6)	2 (4.5)	0.84

*IQR* inter quartile range, *SAPS II* Simplified Acute Physiologic Score II, *SOFA* Sepsis Organ Failure Assessment, *ICU* intensive care unit

^a^31 missing values

^b^Defined by Knaus definition [17]

^c^Anticipated decision of no escalation of life-sustaining intervention before this need arises

Overall, 10/12 (83 %) of the centers had an acute geriatric unit with fixed beds, and 16/176 (14 %) patients >90 years old were transferred in these units after ICU discharge.

## Discussion

In a selected population of very elderly patients, this French multicenter study showed that intensity and monitoring of treatment were quite similar between octogenarian (80–90 years) and nonagenarian patients (>90 years), except for lower use of invasive arterial monitoring and of RRT and overuse of urinary catheter. ICU and hospital mortality were not significantly different.

Very elderly patient population will greatly increase in the future, and one of the tough challenges of intensivists will be to adapt resources to benefit of care [1]. While the rate of very elderly patients admissions clearly increased among hospital admissions [1], overall ICU admissions rate of patients aged 80 and over remained stable, around 15 % in Europe: France 18 % [7], Netherlands 13.8 % [22], and Italy 19.2 % [23] as worldwide: Australia and New Zeeland 13 % [9]. This stable rate was probably explained by a stringent triage [24, 25]. Two studies, each comparing practices to 10 years apart, reported similarly that age >85 remained an independent factor hampering ICU admission [10, 11].

Few studies reported the percentage of nonagenarians in ICU admissions. In our study, nonagenarians represented around 8 % of ICU admissions, higher than the rates observed in a Brazilian [13], Norway [26], or French study [6] 3, 2.5, or 1 %, respectively. The percentage of patients aged 85 years and over represented only 3.4 % in the Eldicus study [25]. As shown in a previous study [27], our study showed that, after having submitted nonagenarian’s patients to a stringent selection process, ICU physicians provided similar treatment as to octogenarian patients, namely ventilation procedures, and vasoactive drugs administration, except for the use of RRT for nonagenarians. Interestingly, this was in agreement with very elderly’s wishes [12]. Intensity of treatment provided to the very elderly has changed overtime. In the last decade, age could be an obstacle for delivering treatment for acute coronary disease [28, 29] or ICU treatment [15]. In a more recent period, Lerolle et al. [5], in a single-center study over a decade, showed that selecting good candidates (mean age: 83 years) with benefit of ICU treatment was associated with improvement of mortality rates, even if they were severely ill at ICU admission, thus requesting greater intensity of treatment (vasoactive drugs and RRT). In a subgroup analysis by 5-year age groups, Andersen et al. [26] reported significant lower time on and use of mechanical ventilator support among the 738 patients of the nonagenarian subgroup compared with other age groups. Improving the process selection is difficult [10, 30–33]; recently, evaluation of frailty [34] was added to the making decision process [35, 36]. Selecting better candidates for ICU admission will be an ongoing process: Will a 90-year-old person in 2030 be as frail and thus have the same risk of ICU admission as in 2015? The global progress in medicine will lead to admit more healthy very elderly in ICU, and the results of ICU treatment of very elderly will probably improve. Of note, scarce studies of trauma centenarians are available [37].

Transferring a patient to a geriatric unit for a geriatric assessment after ICU discharge did not improve hospital mortality. A geriatric assessment is “a combination of a multidimensional interdisciplinary approach and diagnostic process focused on determining in a frail old person, medical, psychological and functional capacity in order to develop a coordinated plan for treatment and recovery” [38]. The benefit of geriatric assessment seems to be more in the post-hospital period. A review of 22 randomized trials having included 10,315 patients showed that geriatric assessment was more often associated with being alive in their own home 1 year after hospital discharge [39]. Our study reported that only 14 % of patients were transferred to a geriatric unit. Due to the retrospective analysis of the study and the long period of inclusion, we were not able to report whether these patients have benefit of a geriatric assessment. Further studies involving more patients and using a randomized process at ICU discharge are needed to explore this point.

One of our study strengths was to include a control group of youngest patients, i.e., octogenarians, to analyze comparatively the intensity of treatment and outcome.

This study has also several limitations. First, our study described practices in France and may not be extrapolated in other countries. Second, the main limitation of the study was the lack of follow-up regarding the outcome of the post-intensive care syndrome [40], namely physical, cognitive, or mental sequelae which funded the benefit of ICU admission. These outcomes were not in our database, and their specific research was difficult due to the high number of female, registered under different names (maiden name or married name) in the hospital register and in the appropriate register death office. Third, our database did not contain measurement of frailty index, which is now used to better describe ICU and hospital outcome [4]. Fifth by fourth, the study was on a 15-year period with possible changes in selection and care of elderly people. Sixth by fifth, age being a continuous variable, using it to compare groups for outcome may appear artificial.

In conclusion, this multicenter study reported that nonagenarians represented a small part of ICU patients. When admitted to ICU, these highly selected patients received similar life-sustaining treatments as octogenarians, except RRT and invasive monitoring for blood pressure. Hospital mortality was not influenced by post-ICU care. Further research is needed to elucidate recovery in this nonagenarian population.

## Additional file

10.1186/s13613-016-0133-9 Evolution of nonagenarians admissions during the study period.
