# Supplementary material for: Treatment intensity and outcome of nonagenarians selected for admission in ICUs: a multicenter study of the Outcomerea Research Group
Source: Ann Intensive Care. 2016 Apr 14;6:31. doi: 10.1186/s13613-016-0133-9 (PMC4830777; doi:10.1186/s13613-016-0133-9)

**Treatment intensity and outcome of nonagenarians selected for admission in ICUs: a multicenter study of the Outcomerea research group**

Maité Garrouste-Orgeas,, Stéphane Ruckly, Charles Grégoire, Anne-Sylvie Dumesnil, Cécile Pommier, Samir Jamali,, Dany Golgran-Toledano, Carole Schwebel, Christophe C’lech,, Lilia Soufir, Muriel Fartoukh, Guillaume Marcotte, Laurent Argaud, Bruno Verdière, Michael Darmon,, Elie Azoulay, Jean-François Timsit,

**ONLINE MATERIAL**

**Figure S1**. Evolution of nonagenarians admissions during the study period.


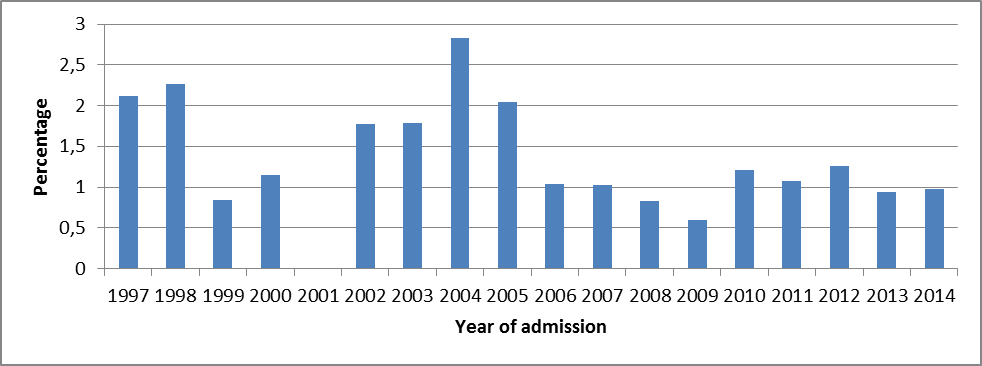

Supplement: Supplementary file 1 — 10.1186/s13613-016-0133-9 Evolution of nonagenarians admissions during the study period. [file 13613_2016_133_MOESM1_ESM.doc]
